# Controlling Nutritional Status (CONUT) score is a prognostic factor in patients with resected breast cancer

**DOI:** 10.1038/s41598-020-63610-7

**Published:** 2020-04-20

**Authors:** Wen Li, Min Li, Ting Wang, Guangzhi Ma, Yunfu Deng, Dan Pu, Zhenkun Liu, Qiang Wu, Xuejuan Liu, Qinghua Zhou

**Affiliations:** 1 Lung Cancer Center, West China Hospital, Sichuan University, Chengdu, Sichuan 610041 P.R. China; 2 Department of Cancer, The People’s Hospital of Yuechi, Guang’an, Sichuan 638300 P.R. China; 3 Department of Breast Surgery, West China Hospital of Sichuan University, Chengdu, Sichuan 610041 P.R. China

**Keywords:** Cancer, Surgical oncology

## Abstract

The present study aimed to determine the correlation between controlling nutritional status (CONUT) and prognosis in resected breast cancer patients. Totally, 861 breast cancer patients with surgical resection in West China Hospital of Sichuan University between 2007 and 2010 were included. The relationship between CONUT and various clinicopathological factors as well as prognosis was evaluated. The results showed that the optimal cutoff value for CONUT to predict the 5-year survival was 3 and CONUT had a higher area under the ROC curve (AUC) for 5-year disease free survival (DFS) and overall survival (OS) prediction compared with the neutrophil lymphocyte ratio (NLR) and prognostic nutritional index (PNI). High CONUT was significantly correlated with older age, lymph node involvement, advanced T-stage, and surgery type. In the multivariate analysis, CONUT-high patients had worse DFS and OS, when compared with CONUT-low patients. In conclusion, preoperative CONUT is a useful marker for predicting long term outcomes in breast cancer patients after curative resection.

## Introduction

Breast cancer is one of the most commonly diagnosed malignancies in women worldwide^1^. Although surgery is the main treatment for breast cancer, its clinical course remains unsatisfactory since an appreciable part of patients develop local recurrence or distal metastasis after resection^2^. Hence, it is vital to find out potential biomarkers to accurately predict the prognosis and provide comprehensive information for selecting appropriate treatment strategies.

It has been identified that the cancer prognosis is, to some extent, related to host status, including nutrition or inflammation^3^. Besides, poor nutritional condition may be correlated with the metabolic elevation and the immune-compromised status in cancer patients^4,5^. Previous studies have reported that preoperative nutritional status, including albumin, is related with the prognosis in several malignancies^4,6^. Immune status is also correlated with tumor formation and recurrence^7,8^. Many indicators, including blood neutrophil, lymphocyte, monocyte, platelet count, neutrophilocyte-to-lymphocyte ratio (NLR), derived neutrophilcyte-to-lymphocyte ratio (dNLR), lymphocyte-to-monocyte ratio (LMR) and platelet-to-lymphocyte ratio (PLR), have been reported to be prognostic predictors in various cancers^9–13^. A meta-analysis showed that the LMR was significantly associated with long term outcomes in colorectal cancer^14,15^. What is more, it has also been verified that a platelet and lymphocyte-to-monocyte ratio (COP-LMR) is a novel prognosis predictor in lung cancer^16^.

The prognostic nutritional index (PNI), which consists of serum albumin concentration and total lymphocyte count, is used to assess the perioperative immunonutritional status and surgical risk for patients^5^. It has been reported that the PNI could predict postoperative complications including the intra-abdominal abscess, postoperative cardiovascular disease and pulmonary disease, pleural effusion, ascites, urinary tract infection, intraperitoneal and subcutaneous bleeding, inflammation of the intestine, obstruction of the intestine, pancreatic fistula, lymphorrhea, and numbness of limbs in patients with colorectal cancer^17^. Furthermore, it is corroborated to be a prognostic factor in various tumors, including breast cancer^17–21^. Based on these findings, a more comprehensive scoring system, controlling nutritional status (CONUT), consisting of serum albumin, cholesterol levels and lymphocyte count in peripheral blood, is yet to be proposed to assess patient nutritional status. Similar to PNI, CONUT could easily be calculated from blood examination data. Recently, it has been shown that CONUT is an independent prognostic marker in malignant pleural mesothelioma^22^, resected lung squamous cell carcinoma^23^, gastric cancer^4^, and head and neck cancer^24^. However, its role in breast cancer has not been reported. To our knowledge, we firstly attempted to assess the prognostic significance of CONUT in breast cancer patients who received curative resection based on a large study.

## Materials and Methods

### Patients and follow-up

A total of 1,364 breast cancer patients who received surgical resection from 2007 to 2010 in West China Hospital of Sichuan University were recruited (Supplementary Dataset 1). The complete preoperative blood cell count was procured within seven days before surgery. The exclusion criteria were as follows: (1) patients who received chemotherapy or radiotherapy before the surgery; (2) patients with inflammatory disease or autoimmune disease; (3) patients who lacked detailed clinicopathological information; (4) male breast cancer patients. Finally, 861 cases were included in the present retrospective study. All the patients were followed up every three months in the first three years, every six months for five years, and annually within 6–10 years after the operation. Clinical check-up, laboratory examination and radiological assessment were included in the follow-up investigations.

### Pathology methods and molecular subtypes

Estrogen receptor (ER), progesterone receptor (PR), human epidermal growth factor receptor 2 (HER2) statuses and Ki67 expression were assessed by immunohistochemical staining. The monoclonal ER antibody (clone SP1; Ventana, Tucson, AZ, USA), monoclonal PR (clone 1E2; Ventana), Ki-67 (clone 30–9; Ventana) and HER2 (clone 4B5; Roche, Sandhofer, Mannheim, Germany) were used. Positive ER or PR was defined as ≥1% of immunoreactive tumor cell nuclei, according to the American Society of Clinical Oncology and College of American Pathologists Guideline Recommendations in 2010. The cutoff value for Ki-67 was defined as ≥14%. As for HER-2, 0 or 1+ was negative, while 3+ was reported as positive. Fluorescence *in situ* hybridization (FISH) was performed in case of a 2+ level of staining.

The molecular subtypes were classified as Luminal A (ER+ and/or PR+, HER2−, Ki-67 < 14), Luminal B (ER+ and/or PR+, HER2+ and/or HER2-, any Ki-67), HER2-enriched (ER−, PR−, HER2+, any Ki-67), and triple-negative (ER−, PR−, HER2−, any Ki-67) breast cancer (TNBC).

### Ethical approval and consent to participate

The study has been approved by the Institutional Ethical and Scientific Committee of West China Hospital of Sichuan University. Written informed consent was obtained from all participants in accordance with the policies of the committee. All methods applied within the study were performed according to the approved guidelines.

### CONUT score and other scoring systems

The blood samples were investigated in one week before surgery. According to previous studies, the CONUT score was obtained based on serum albumin concentration, cholesterol level, and lymphocyte count (Table 1). The PNI was calculated by utilizing the following formula: 10 × the serum albumin value (g/dl) + 0.005 × the total lymphocyte count in peripheral blood (per mm^3^). The neutrophil-to-lymphocyte ratio was determined as the absolute neutrophil count divided by the absolute lymphocyte count.

**Table 1:** The CONUT scoring system.

Parameters	Normal	Light	Moderate	Severe
Serum albumin (g/dL)	≥3.50	3.00–3.49	2.50–2.99	<2.50
score	0	2	4	6
Total lymphocyte count	≥1600	1200–1599	800–1199	<800
score	0	1	2	3
Total cholesterol (mg/dL)	>180	140–180	100–139	<100
score	0	1	2	3
CONUT score (total)	0–1	2–4	5–8	9–12
Assessment	Normal	Light	Moderate	Severe

### Determination of the cutoff value

The receiver operating characteristic (ROC) curve was used to assess the sensitivity and specificity for 5-year survival. In addition, the Youden index was calculated to choose the best cutoff value.

### Statistical analysis

OS was defined as the interval from diagnoses to death of any cause or last follow-up, whichever occurred first. DFS was calculated from the time of diagnoses to the first observation of recurrence or last follow-up without evidence of recurrence. The association between clinicopathological factors and CONUT was analyzed by *X*^2^-test. Variable was assessed on the univariate analysis, and then was calculated on the multivariable Cox proportion analysis if it was statistically significant. All statistical analyses were conducted by the SPSS (version 20.0) software pack (SPSS Inc., Chicago, IL, USA). *P* < 0.05 was statistically significant.

## Results

### ROC analysis

**Figure 1:**
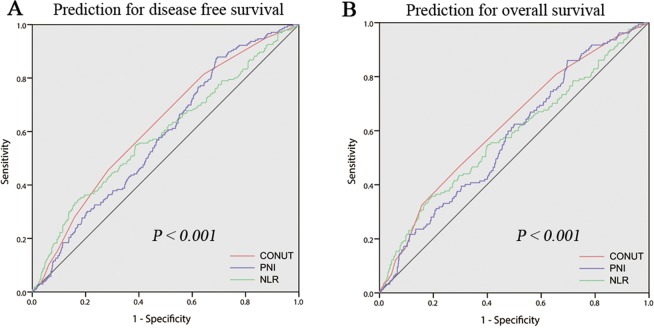
The ROC curves of CONUT, NLR and PNI for predicting DFS (**A**) and OS (**B**).

Using the 5-year survival as an endpoint, 3 was considered to be the best cutoff value for CONUT since the corresponding Youden index was maximal. The sensitivity and specificity for OS were 81.6% and of 35.7%, respectively (Fig. 1A,B). All the patients were classified into CONUT-low group (≤2) and CONUT-high group (≥3).

### Comparison of CONUT with NLR or PNI

The prognostic accuracies of CONUT, PNI and NLR were explored by the AUC of the ROC curve for predicting the 5-year DFS and OS (Fig. 1A,B). The AUCs of CONUT, NLR and PNI for DFS were 0.622 (95% CI: 0.580–0.665), 0.590 (95% CI: 0.543–0.636), and 0.581 (95% CI: 0.539–0.624), respectively, while the AUCs of CONUT, NLR and PNI for OS were 0.621 (95% CI: 0.573–0.669), 0.579 (95% CI: 0.527–0.631), and 0.577 (95% CI: 0.530–0.625), respectively.

### The correlation between CONUT and clinicopathological factors

Among the 861 breast cancer patients included in the present study, 223 patients were classified as luminal A subtype (25.9%), 407 patients were Luminal B subtype (47.3%), 135 patients were HER2 subtype (15.7%), and 96 patients were TNBC subtype (11.1%). The median age was 55 years old, with a median follow-up of 61.7 months. 206 patients developed tumor relapsed and154 patients died. The clinical and pathologic characteristics of the 861 patients in the present study were presented in Table 2. A high CONUT was significantly related with age, lymph node involvement, advanced T-stage and surgery type, but not related with Ki-67 status, high tumor grade, ER status, PR status, or HER2 over expression.

**Table 2:** Patient and tumor characteristics by CONUT group.

	Total	CONUT ≤ 2	CONUT ≥ 3	P
**Age**		581	280	0.003
≤ 40	211 (24.5%)	160 (27.5%)	51 (18.2%)	
> 40	650 (75.5%)	421 (72.5%)	229 (81.8%)	
**ER**				0.456
+	538 (62.5%)	368 (63.3%)	170 (60.7%)	
−	323 (37.5%)	213 (36.7%)	110 (39.3%)	
**PR**				0.505
+	396 (46.2%)	264 (45.4%)	134 (47.9%)	
−	465 (53.8%)	317 (54.3%)	146 (52.1%)	
**HER2**				0.253
+	198 (23.0%)	127 (21.9%)	71 (25.4%)	
−	663 (77.0%)	454 (78.1%)	209 (74.6%)	
**Ki-67 status**				0.246
+	568 (65.2%)	358 (63.8%)	190 (67.9%)	
−	293 (34.8%)	203 (36.2%)	90 (32.1%)	
**pT Stage**				0.003
1	287 (33.3%)	209 (37.3%)	78 (26.0%)	
2	449 (52.1%)	283 (50.4%)	166 (55.3%)	
3	91 (10.6%)	49 (8.7%)	42 (14.0%)	
4	34 (3.9%)	20 (3.6%)	14 (4.7%)	
**pN Stage**				P < 0.001
0	370 (43.0%)	278 (47.9%)	92 (32.7%)	
1	309 (35.9%)	203 (35.0%)	106 (37.7%)	
2	130 (15.1%)	69 (11.9%)	61 (21.7%)	
3	52 (6.0%)	30 (5.2%)	22 (7.8%)	
**Molecular subtype**				0.095
Luminal A	223 (25.9%)	162 (27.9%)	61 (21.8%)	
Luminal B	407 (47.3%)	262 (45.1%)	145 (51.8%)	
HER2-enriched	135 (15.7%)	87 (15.0%)	48 (17.1%)	
TNBC	96 (11.1%)	70 (12.0%)	26 (9.3%)	
**Histological grade**				0.227
I-II	585 (67.9%)	387 (66.6%)	198 (70.7%)	
III	276 (32.1%)	194 (33.4%)	82 (29.3%)	
**Surgery type**				0.041
Mastectomy	688 (79.9%)	453 (78%)	235 (83.9%)	
BCS	173 (20.1%)	128 (22%)	45 (16.1%)	
**Chemotherapy**				0.057
Yes	606 (70.4%)	397 (68.3%)	209 (74.6%)	
No	225 (29.6%)	184 (31.7%)	71 (25.4%)	
**Hormonal therapy**				0.233
Yes	655 (76.1%)	435 (74.9%)	220 (78.6%)	
No	206 (26.9%)	146 (25.1%)	60 (21.4%)	
**Radiotherapy**				0.320
Yes	393 (45.6%)	121 (43.2%)	272 (46.8%)	
No	468 (54.4%)	159 (56.8%)	309 (53.2%)	
**Target therapy**				0.114
Yes	125 (14.5%)	92 (15.8%)	33 (11.8%)	
No	736 (85.5%)	489 (84.2%)	247 (88.2%)	

### Correlations of the CONUT score with survival

**Figure 2:**
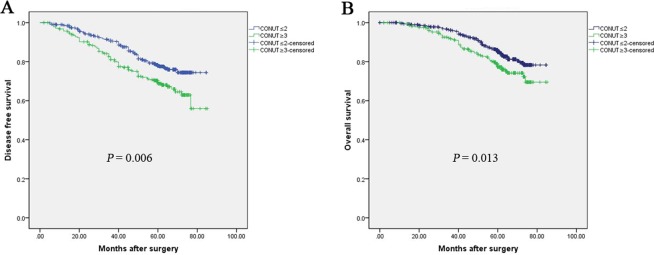
Kaplan-Meier survival analyses of the correlation between CONUT and survival among breast cancer patients: DFS (**A**) and OS (**B**).

**Figure 3:**
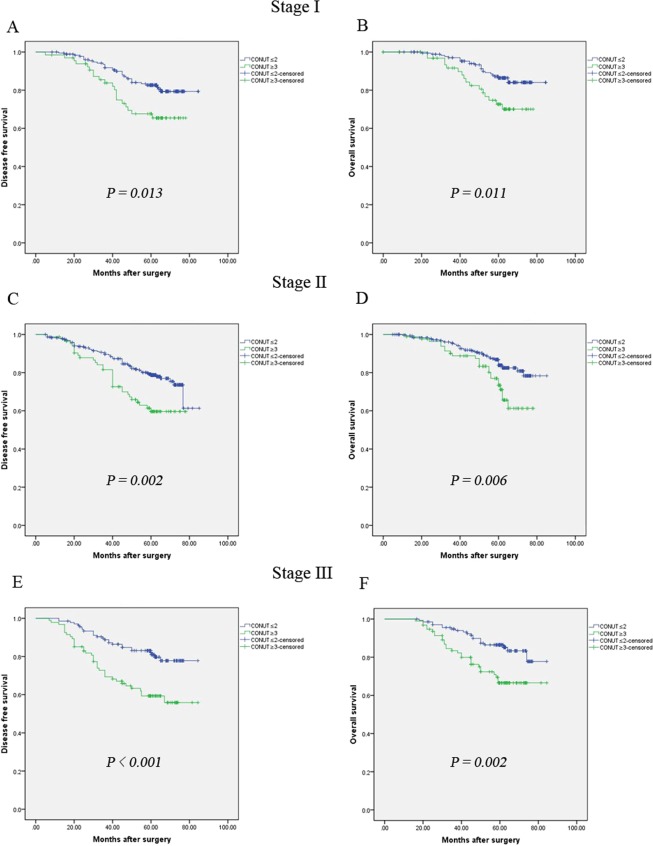
Kaplan-Meier survival analyses of DFS and OS, according to CONUT, among patients in the stage I, stage II and stage III subgroups.

The results revealed that a high CONUT was a poor prognostic factor for both DFS and OS in breast cancer patients. The 5-year OS rates were 68.7% in the COUNT-high group and 77.9% in the COUNT-low group (*P* = 0.013, Fig. 2A). In addition, the 5-year DFS rates were 76.6% in the COUNT-high group and 84.6% in the COUNT-low group (*P* = 0.006, Fig. 2B). After adjusting for p-stage, CONUT-high was still associated with worse DFS and OS in these three subgroups (Fig. 3A–F).

In the univariate analysis, high CONUT, patient age, PR status, tumor grade, T-stage, lymph node involvement and histological grade were related with DFS and OS. In the multivariate analysis, high CONUT (*P* = 0.07), patient age (*P* = 0.037), PR status (*P* = 0.041), tumor grade (*P* = 0.009), T-stage (*P* = 0.001) and lymph node involvement post-surgery (*P* = 0.002) were independent predictors of DFS, while high CONUT (*P* = 0.027), patient age (*P* = 0.042), tumor grade (*P* = 0.003), T-stage (*P* = 0.031) and lymph nodes (*P* = 0.002) were correlated with OS (Tables 3 and 4).

**Table 3:** Analyses regarding the prognostic factors for disease free survival.

	Univariate analysis		Multivariate analysis	
	HR (95% CI)	P value	HR (95% CI)	P value
**CONUT**	1.486 (1.118–1.975)	0.006	1.548 (1.127–2.125)	0.07
CONUT ≤ 2				
CONUT ≥ 3				
**Patient age**	0.673 (0.497–0.910)	0.01	0.705 (0.507–0.980)	0.037
≤40				
>40				
**ER**	1.127 (0.812–1.564)	0.475		
+				
−				
**PR**	0.715 (0.536–0.954)	0.022	0.737 (0.550–0.988)	0.041
−				
+				
**HER2**	0.874 (0.630–1.211)	0.418		
−				
+				
**Ki-67 status**	1.276 (0.930–1.749)	0.131		
−				
+				
**pT Stage**	1.404 (1.234–1.597)	<0.001	1.307 (1.123–1.522)	0.001
1				
2				
3				
4				
**pN Stage**	1.518 (1.307–1.764)	<0.001	1.333 (1.098–1.599)	0.002
0				
1				
2				
3				
**Molecular subtype**	0.983 (0.846–1.142)	0.824		
Luminal A				
Luminal B				
HER2-enriched				
TNBC				
**Histological grade**	1.587 (1.723–2.15)	<0.001	1.476 (1.101–1.979)	0.009
I-II				
III				
**Surgery type**	1.112 (0.823–1.486)	0.456		
Mastectomy				
BCS				
**Chemotherapy**	0.931 (0.756–1.268)	0.631		
No				
Yes				
**Hormone therapy**	0.867 (0.754–1.625)	0.374		
No				
Yes				
**Radiotherapy**	1.09 (0.826–1.468)	0.561		
No				
Yes				
**Target therapy**	1.159 (0.876–1.542)	0.32		
No				
Yes

**Table 4:** Analyses regarding the prognostic factors for overall survival.

	Univariate analysis		Multivariate analysis	
	HR (95% CI)	P value	HR (95% CI)	P value
**CONUT**	1.514 (1.108–2.198)	0.013	1.220 (1.023–1.455)	0.027
CONUT ≤ 2				
CONUT ≥ 3				
**Patient age**	0.669 (0.472–0.947)	0.023	0.673 (0.460–0.985)	0.042
≤40				
>40				
**ER**	1.346 (0.906–1.999)	0.141		
−				
+				
**PR**	0. 684 (0.490–0.956)	0.026	0.721 (0.502–1.034)	0.076
−				
+				
**HER2**	0.879 (0.605–1.279)	0.501		
+				
−				
**Ki-67 status**	1.161 (0.811–1.662)	0.415		
+				
−				
**pT Stage**	1.452 (1.257–1.678)	<0.001	1.219 (1.017–1.462)	0.031
1				
2				
3				
4				
**pN Stage**	1.582 (1.338–1.870)	<0.001	1.401 (1.135–1.730)	0.002
0				
1				
2				
3				
**Molecular subtype**	0.901 (0.854–1.076)	0.250		
Luminal A				
Luminal B				
HER2-enriched				
TNBC				
**Histological grade**	1.683 (1.274–1.792)	<0.001	1.635 (1.193–2.381)	0.003
I-II				
III				
**Surgery type**	1.077 (0.821–1.46)	0.62		
Mastectomy				
BCS				
**Chemotherapy**	0.932 (0.721–1.236)	0.718		
Yes				
No				
**Hormone therapy**	0.905 (0.678–1.205)	0.462		
Yes				
No				
**Radiotherapy**	1.036 (0.774–1.387)	0.812		
Yes				
No				
**Target therapy**	1.119 (0.836–1.498)	0.45		
Yes				
No				

Since there are four molecular subtypes for breast cancer, the prognostic value of CONUT was subsequently analyzed in these four subgroups. The results revealed obvious associations of high CONUT score and worse outcomes in the luminal B subgroup (Tables 5 and 6).

**Table 5:** Analyses results of CONUT for the prediction of disease free survival in different breast cancer subtypes.

	Univariate analysis		Multivariate analysis	
	HR (95% CI)	p value	HR (95% CI)	p value
**Luminal A**	1.516 (0.781–2.945)	0.219		
CONUT ≤ 2				
CONUT ≥ 3				
**Luminal B**	1.704 (1.153–2.519)	0.007	1.604(1.065–2.414)	0.024
CONUT ≤ 2				
CONUT ≥ 3				
**TNBC**	2.272 (1.091–4.731)	0.028	1.423 (0.917–2.209)	0.116
CONUT ≤ 2				
CONUT ≥ 3				
**HER2-enriched**	2.398 (1.076–5.346)	0.032	1.925 (0.715–5.180)	0.195
CONUT ≤ 2				
CONUT ≥ 3

**Table 6:** Analyses results of CONUT for the prediction of overall survival in different breast cancer subtypes.

	Univariate analysis		Multivariate analysis	
	HR (95% CI)	p value	HR (95% CI)	p value
**Luminal A**	1.403 (0.661–2.980)	0.378		
CONUT ≤ 2				
CONUT ≥ 3				
**Luminal B**	2.213 (1.388–3.530)	0.001	1.878 (1.154–3.055)	0.01
CONUT ≤ 2				
CONUT ≥ 3				
**TNBC**	1.429 (0.662–3.081)	0.363		
CONUT ≤ 2				
CONUT ≥ 3				
**HER2-enriched**	2.542 (1.067–6.465)	0.056		
CONUT ≤ 2				
CONUT ≥ 3				

## Discussion

Studies have recently demonstrated the impact of CONUT on prognosis in several malignancies. In the present study, the prognostic value of CONUT in female breast cancer was initially assessed. Our results showed that CONUT was more accurate in prognosis prediction, when compared with previously reported prognostic scoring systems, PNI or NLR. Furthermore, the results indicated that CONUT was associated with age, tumor size and invasion. Importantly, CONUT independently predicted the prognosis of breast cancer patients, regardless of the tumor stage. Patients with high CONUT predicted the shorter DFS and OS, when compared with patients with low CONUT. Overall, these results suggested that CONUT might be a prognostic factor in breast cancer patients undergoing potentially curative resection. To our knowledge, our study firstly demonstrated the association between preoperative CONUT and clinicopathological factors or survival in breast cancer patients who underwent resection.

CONUT was originally reported as an efficient tool for the early detection and continuous control of hospital undernutrition^25^. Forward studies have demonstrated that CONUT has a prognostic impact on patients with severely decompensated acute heart failure^26,27^. Since CONUT was based on the serum albumin level, total cholesterol level and total lymphocyte count, the CONUT score could reflect the malnutrition and systemic inflammation status. Besides, tumor progression and treatment tolerance have been revealed to be closely correlated with the nutritional and inflammation status. Thus, CONUT could theoretically be a comprehensive prognostic factor. In the present study, the results show that CONUT is associated with both DFS and OS for all the included patients, and a high CONUT score might be associated with a poor prognosis.

PNI and NLR are both reported scoring systems for the evaluation of the general condition of patients and have been demonstrated to be related with cancer survival, including breast cancer^2,28,29^. Comparisons between CONUT and PNI, previous results suggested that CONUT tended to be more superior to the PNI scoring systems for the prediction of survival in various cancer patients. In the present study, our results suggested that CONUT was proved to be superior to both PNI and LNR for the prognosis prediction in resected breast cancer patients.

Among the three components of CONUT, serum albumin concentration is the most important parameter, which is twice the weight of the other two. It is a reliable indicator not only for nutritional status but also for systemic inflammation^30,31^. Studies demonstrated that low serum albumin was associated with poor survival and increased risk of cancer-related death in breast cancer patients^32,33^. Besides, pro-inflammatory cytokines (such as IL-6 or TNF-α) and CRP could also cause decreased serum albumin concentration and modulate albumin synthesis via hepatocytes^34–36^. As cholesterol plays a crucial role in forming cell membranes, cholesterol is related with numerous biochemical pathways which are potentially correlated immune response besides tumorigenesis^37–39^. It has also been reported that low cholesterol level is correlated with poor prognosis in various malignancies as the cholesterol may affect the caloric intake and cell membrane formation^40,41^. What is more, low peripheral lymphocyte count is an indicator for the inadequate host immune response and is correlated to undesirable prognosis in various cancers, including breast cancer^9,42,43^. Thus, the combination of these three parameters could integrate the accuracy of each parameter to assess for the general condition.

## Conclusion

The present study indicates that CONUT is a useful prognostic factor for breast cancer patients undergoing curative resection, and a high CONUT score might be associated with a poor prognosis.

## Supplementary information

Supplementary Dataset 1.

## Data Availability

All data generated or analyzed during this study are included in this published article.
